# Insecticide-Mediated Shift in Ecological Dominance between Two Competing Species of Grain Beetles

**DOI:** 10.1371/journal.pone.0100990

**Published:** 2014-06-24

**Authors:** Erick Maurício G. Cordeiro, Alberto S. Corrêa, Raul Narciso C. Guedes

**Affiliations:** 1 Departamento de Entomologia, Universidade Federal de Viçosa, Viçosa, Minas Gerais, Brazil; 2 Departamento de Entomologia e Acarologia Agrícola, Escola Superior de Agricultura “Luiz de Queiroz” (ESALQ), Universidade de São Paulo (USP), Piracicaba, São Paulo, Brazil; Institute of Vegetables and Flowers, Chinese Academy of Agricultural Science, China

## Abstract

Competition is a driving force regulating communities often considered an intermittent phenomenon, difficult to verify and potentially driven by environmental disturbances. Insecticides are agents of environmental disturbance that can potentially change ecological relationships and competitive outcomes, but this subject has seldom been examined. As the co-existing cereal grain beetle species *Sitophilus zeamais* Motschulsky and *Rhyzopertha dominica* F. share a common realized niche, directly competing for the same resources, they were used as models in our study. Intraspecific competition experiments were performed with increasing insect densities and insecticide doses in additive and replacement series using various density combinations of both beetle species maintained on insecticide-free or -sprayed grains. Insecticide-mediated release from competitive stress was not observed in our study of intraspecific competition in grain beetles. The insecticide enhanced the effect of insect density, particularly for the maize weevil *S. zeamais*, further impairing population growth at high densities. Therefore, insecticide susceptibility increased with intraspecific competition favoring insecticide efficacy. However, the effect of insecticide exposure on competitive interaction extends beyond intraspecific competition, affecting interspecific competition as well. *Sitophilus zeamais* was the dominant species when in interspecific competition prevailing in natural conditions (without insecticide exposure), but the dominance and species prevalence shifted from *S. zeamais* to *R. dominica* under insecticide exposure. Therefore, high conspecific densities favored insecticide efficacy, but the strength of the relationship differs with the species. In addition, the insecticide mediated a shift in species dominance and competition outcome indicating that insecticides are relevant mediators of species interaction, potentially influencing community composition and raising management concerns as potential cause of secondary pest outbreaks.

## Introduction

Competition, as a mutually negative interaction between two species sharing the same guild or trophic level, results in reduced abundance or in a decrease in fitness components of the competing species, potentially regulating animal communities [1]–[4]. The phenomenon is often considered intermittent and difficult to verify, producing skepticism and, thus, controversy about its role in shaping communities [3], [5]–[7]. Small change(s) in the shared (realized) niche between two species may compromise the optimal development of one of them, altering the outcome of competition and potentially determining a shift of the prevailing or dominant species [8]–[13].

Environmental disturbances, either natural or artificial, can affect ecological interactions and produce changes in the (realized) niche shared by competing species [11]–[14]. Pesticides, particularly insecticides, are seasonal and intermittent agents of environmental disturbance that may alter ecological relationships, analogous to the effects of a flood or storm in a natural community [13]–[17]. Insect species, particularly insect pest populations, can respond rapidly to such disturbances [13], [14], [16]–[19]. Recovery, if possible, can require more time than that needed to allow the degradation of the insecticide to reduce the contaminant residue to the background level. Accordingly, the recovery time can depend on the ability of the species to survive and develop in the contaminated environment.

The lethal acute effect of insecticides is obviously the chief concern of toxicological and management studies. Far less attention has been devoted to sublethal effects despite an apparent shift in attention that has focused primarily on beneficial insects [18]–[21]. The ability to grow during the intermediate and final stages of disturbance may essentially define the outcome of ecological interactions in insecticide-contaminated environments. However, few studies have explored the seasonal variability of competition and the importance of disruptive events in competition, particularly if an insecticide is the disruptive agent [22]–[24]. The only few studies available in arthropods are rather recent and focused on pointed differences in the occurrence of competing species between area subjected (or not) to insecticide applications, as with whiteflies [25] and leafminer flies [26], and just a single study with mosquitoes explored density-dependence on the competition outcome, but without considering the dose-dependent effect of the insecticide used [27].

Insecticides affect insect survival and may also affect a series of life history and behavioral traits of the exposed individuals of a given species [18]–[20], [23]. As different species exhibit innate differences in susceptibility and the likelihood of exposure, it is probable that they will show differing responses to insecticide stress disturbance. This consideration is, most likely, more important for sublethal levels of exposure because they prevail in the field, not only because of the degradation of insecticides in the environment but also because the lethal effects of modern insecticides are targeted at specific pest species, which is(are) the target(s) of control. Most likely, however, these insecticides also exhibit sublethal effects on a broader range of co-occurring species in the contaminated area.

Beetles of stored cereal grains, such as the maize weevil *Sitophilus zeamais* Motschulsky (Coleoptera: Curculionidae) and the lesser grain borer *Ryzopertha dominica* F. (Coleoptera: Bostrichidae), are important pest species that spend their immature stages within a single grain. A single grain can receive multiple eggs and/or larvae, thus increasing the competition among larvae within the grain [28]–[32]. Both of these grain beetle species co-occur and compete for the same resource (i.e., cereal grains), which is treated with insecticides as a management measure to minimize potential losses [33], [34]. These traits make these species potential models for intra- and interspecies competition under insecticide exposure as an environmental stress agent. Although this subject is important, it has received very limited attention.

In this study, we performed intraspecific competition experiments with increasing insect densities (of *S. zeamais* and *R. dominica*) and increasing insecticide doses. Interspecific competition between *S. zeamais* and *R. dominica* was assessed through additive and replacement series using different density combinations of both beetle species maintained on insecticide-free or insecticide-sprayed grains. As the insecticide used, the organophosphate fenitrothion, is recommended for maize grain protection, the chief expected result was an alleviation of intra- and interspecific competition, allowing better species coexistence. However, the dominant species shifted from *S. zeamais*, the prevailing species without insecticide exposure, to *R. dominica* under insecticide exposure. Such a shift in species dominance and competition outcome indicates that insecticides are relevant mediators of species interaction, with lasting effects potentially influencing community composition and raising management concerns, including their potential mediation of the occurrence of secondary pest outbreaks.

## Materials and Methods

### Ethics Statement

The study did not involve any endangered or protected species. The insect species studied are pest species maintained in laboratory, where the experiments were carried out. The laboratory colonies were initially established from over 200 field-collected individuals, for which no specific permission was required at the time.

### Insects

A strain of the maize weevil *Sitophilus zeamais* and a strain of the lesser grain borer *Rhyzopertha dominica* were used in the experiments. Both strains were originally collected from stored maize in Viçosa county (State of Minas Gerais, Brazil) in the early 2000's and have since been maintained in the laboratory without insecticide exposure. Both populations are susceptible to fenitrothion, and this susceptibility is periodically checked. The populations are maintained in glass containers (1.5 L) within growth chambers (28±2°C, 70±10% relative humidity, 12 h∶12 h photoperiod (D∶L)) and reared on insecticide-free whole maize grains. The competition experiments were conducted under these same environmental conditions.

### Insecticide

The insecticide used as the agent of disturbance in our competition experiments was the organophosphate fenitrothion, used in its commercial formulation registered in Brazil for maize grain protection (500 g a.i./L; emulsifiable concentrate; Sumitomo Chemical do Brasil) [35]. The insecticide solutions (with distilled and deionized water as the solvent) were sprayed at a rate of 1 mL (insecticide) emulsion on 500 g of maize grains (13% humidity) placed in a rotary stainless steel container to homogenize the grain during the application. An artist's air brush (Sagyma SW440A, Yamar Brasil, São Paulo, SP, Brazil) coupled with an air pump (Prismatec 131A Tipo 2VC, Itu, SP, Brazil) was used for insecticide spraying. The spraying was performed at a pressure of 0.7 kgf/cm^2^. The grains were kept in the container after spraying and allowed to dry (the drying process was complete after one hour, and the grains were then removed).

### Intraspecific competition experiments

The single-species (intraspecific) competition experiments were conducted in glass jars (1.5 L) containing 300 g of whole maize grains. Four jars (i.e., replicates) were used for each combination of insect density (50, 100, 150 and 200 unsexed adult insects (two weeks old) per jar), insecticide dose (0.0, 0.2, 0.35, 0.5 and 5.0 ppm a.i.) and species (*S. zeamais* and *R. dominica*), following a three-way factorial arrangement in a completely randomized design. The adult insects were released in each jar, and after 90 days the number of dead and live insects were recorded, as well as the average (adult) insect body mass (in samples of 20 insects per replicated jar) and grain consumption. The instantaneous rate of population growth (r_i_), a robust surrogate estimator of the intrinsic rate of population growth (r_m_) [19], [36], was calculated using the formula r_i_ = [Ln(*N_j_*/*N_i_*)]/Δt, where *N_j_* and *N_i_* are the final and initial number of live insects (in each jar), respectively, and Δt is the duration of the experiment (i.e., 90 days).

A second experiment exploring the effect of crowding on insecticide susceptibility was also conducted by exposing adults of either *S. zeamais* or *R. dominica* to two crowding conditions (low density: 50 insects per jar; high density: 150 insects per jar) and to increasing doses of the insecticide fenitrothion (0.0, 0.2, 0.35, 0.5 and 0.7 ppm a.i.). The methods used were the same as detailed above, with four replicates in a completely randomized design.

### Interspecific competition experiments

Two sets of experiments were conducted to explore the characteristics of interspecific competition between *S. zeamais* and *R. dominica*. The first set followed a replacement series using the following density combinations of *S. zeamais* and *R. dominica*: 200∶0, 150∶50, 100∶100, 50∶150, and 0∶200. The second experiment followed an additive series, placing the two competing species in various even densities (5∶5, 10∶10, 25∶25, 50∶50, 100∶100, 150∶150, 200∶200) and uneven densities (5∶100, 10∶100, 20∶1400, 40∶1400, 60∶800, 150∶1200, 270∶600 (*S. zeamais*∶*R.dominica*)). Both experiments were performed with maize grains sprayed with fenitrothion (0.7 ppm a.i.) and with maize grains without insecticide spraying. The insecticide concentration used was selected based on the results of intraspecific competition because it affected the species interaction significantly without eliminating any species. Four replicated jars were used for each treatment in the first experiment, and a single jar was used for each treatment in the second experiment.

### Statistical analyses

A multivariate analysis of covariance was used to analyze the results of intraspecific competition. The procedure GLM with the MANOVA statement from SAS [37] was used for this analysis. The independent (predictor) variable was insect species, with insect density and insecticide concentration as covariates and the number of dead insects, grain consumption, insect body mass and population growth rate (r_i_) as dependent variables. Subsequent (univariate) analyses of covariance complemented with regression analyses were performed if necessary to recognize the existing differences and trends. The potential relationship between increases in insect density and insecticide susceptibility for *S. zeamais* and *R. dominica* was tested using an analysis of covariance complemented by regression analyses with insecticide dose as the covariate in two densities (independent variable) for each species: low density (50 insects per jar) and high density (150 insects per jar). Population growth rate (r_i_) was the dependent (response) variable tested. The regression analyses were performed using the procedure REG from SAS [37]. Only robust linear relationships (i.e. robust fit of regression curves exhibiting R^2^>0.30) were considered in accordance with the descriptive purpose of this study.

Regression analyses with the initial density of the heterospecific competitor as the independent variable and population growth rate as the dependent variable were performed for the interspecific competition experiments using replacement series (procedure REG [37]). In contrast, for the additive series experiment on interspecific competition, descriptive contour plots were generated describing the population growth rate of *S. zeamais* and *R. dominica* under varying densities of each species with or without insecticide exposure.

## Results

### Intraspecific competition

A multivariate analysis of covariance indicated overall significant effects of grain beetle species, insect density and insecticide dose on the response variables assessed (i.e., number of dead insects, insect body mass, grain consumption and population growth rate) (*P*<0.05). The density-dose, dose-species and density-species interactions were all significant, as well as the individual sources of variation (i.e., species, density and dose) (Wilks' lambda >0.219, F_appr._>3.95, *P*<0.001). A subsequent (univariate) analysis of covariance for each response variable reinforced the trend observed in the multivariate analysis of covariance. Because the number of dead insects and insect body mass were correlated with grain consumption (n = 124, r = 0.70, *P*<0.001; and n = 105, r = 0.51, P<0.001, respectively), which was itself correlated with the population growth rate (r_i_) (n = 124, r = 0.42, *P*<0.001), we will focus on r_i_ because it is a more robust toxicological endpoint and meaningful demographic variable [19], [38].

**Figure 1:**
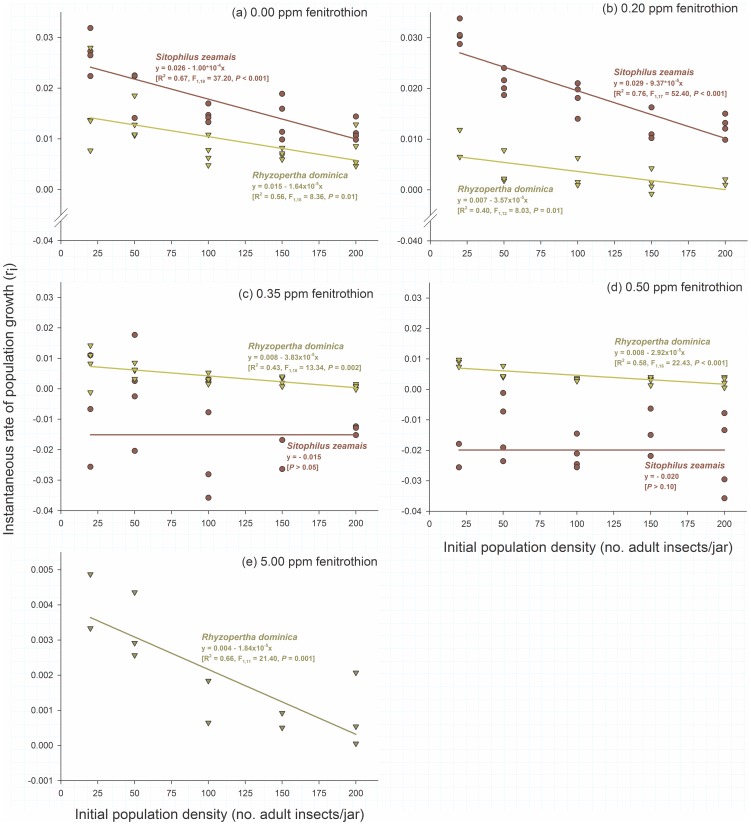
Effect of initial (conspecific) adult density on the population growth of two species of stored grain beetles (*Sitophilus zeamais* and *Rhyzopertha dominica*) reared on maize grains treated with increasing doses of the organophosphate insecticide fenitrothion. Each symbol represents the results of an experimental replicate.

The population growth rate (r_i_) decreased significantly (*P*<0.001) with increased density in both insect species without any insecticide exposure (Fig. 1a). The maize weevil *S. zeamais* prevailed without insecticide application, exhibiting higher population growth than *R. dominica*, but both species were similarly affected by conspecific density (F_1,36_ = 2.45, *P* = 0.13; Fig. 1a). The same trend also occurred under insecticide exposure at a dose of 0.2 ppm (Fig. 1b) but was reversed at 0.35 ppm fenitrothion (Fig. 1c). Fenitrothion at 0.5 ppm compromised the reproduction of *S. zeamais*, resulting in negative population growth and thus preventing intraspecific competition in this species, in contrast with the outcome for *R. dominica* (Fig. 1d). At this insecticide dose, the density of *S. zeamais* did not affect the population growth of this species because no competition occurred. This finding is confirmed by the results at the highest insecticide dose used, 5.0 ppm, where *S. zeamais* showed no survival and only *R. dominica* survived.

**Figure 2:**
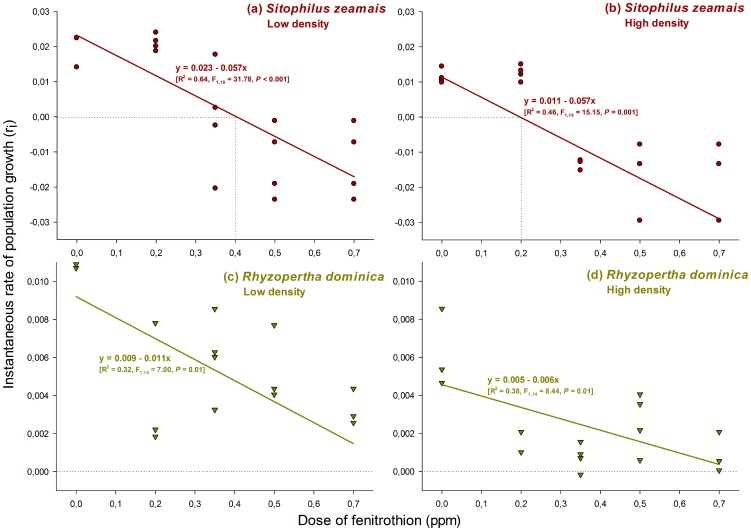
Effect of (conspecific) crowding (low (50 insects/jar) or high (150 insects/jar) initial density) on the population growth of two species of stored grain beetles (*Sitophilus zeamais* and *Rhyzopertha dominica*) reared on maize grains treated with increasing doses of the organophosphate insecticide fenitrothion. Dotted lines indicate the concentration at which population growth is zero (i.e., does not occur). Each symbol represents the results of an experimental replicate.

The effect of crowding on insecticide susceptibility was also assessed. The density-dose interaction was significant for *S. zeamais* (F_4,26_ = 7.27, *P*<0.001), but not for *R. dominica* (F_4,23_ = 1.27, *P* = 0.31). However, *R. dominica* exhibited significant effects of both insect density (F_1,23_ = 8.63, *P* = 0.007) and insecticide dose (F_4,23_ = 5.83, *P* = 0.002). The susceptibility of *S. zeamais* increased approximately 50% under crowded conditions, with zero population growth occurring at 0.4 ppm fenitrothion for insects at low density and at 0.2 ppm fenitrothion for high-density conditions (Fig. 2a, 2b). The susceptibility of *R. dominica* was also negatively affected by crowding, although less drastically than that of *S. zeamais*, with zero growth reached at fenitrothion doses ranging from 0.6–0.7 ppm (Fig. 2c, 2d).

### Interspecific competition

The potential prevalence and competitive dominance of *S. zeamais* over *R. dominica* in maize grains was further tested in direct competition experiments involving co-infestation with both species. The potential shift in such dominance with insecticide exposure was also tested by determining the population growth rate under varying proportions of both co-occurring species in treated and untreated maize grains. The treatments were performed with fenitrothion at 0.7 ppm a.i.

**Figure 3:**
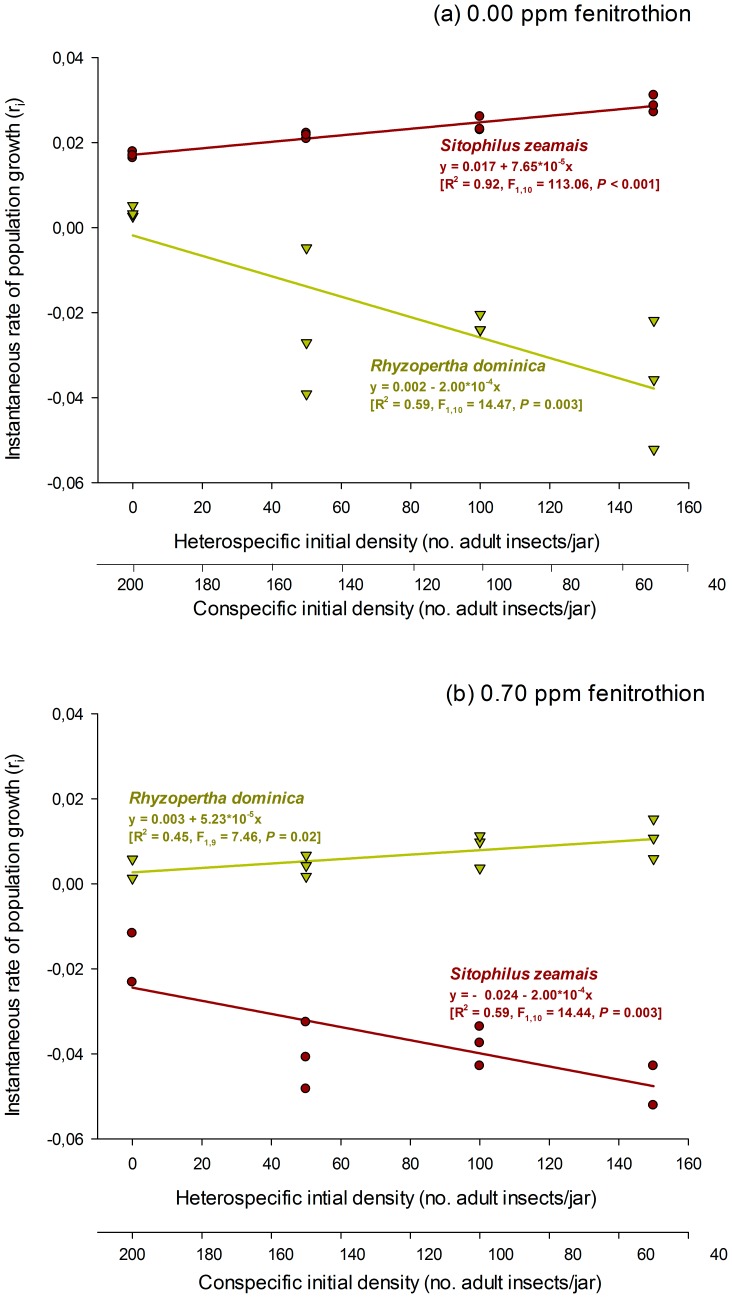
Effect of heterospecific density on the population growth of two species of stored grain beetles (*Sitophilus zeamais* and *Rhyzopertha dominica*) reared on maize grains free of insecticide residue (a) and reared on maize grains treated with 0.7 ppm of the organophosphate insecticide fenitrothion (b). Each symbol represents the results of an experimental replicate.

The increase in heterospecific density in the replacement series without insecticide exposure negatively affected the population growth of *R. dominica* but showed a positive, although more modest, effect on the population growth of *S. zeamais* (Fig. 3a). When the insecticide was introduced into the system, the trends of population growth with increased heterospecific density were reversed, and a shift in competitive dominance occurred (Fig. 3b). The maize weevil *S. zeamais* prevailed without insecticide exposure, producing negative population growth in *R. dominica* and further increasing the maize weevil densities. In contrast, *S. zeamais* was more drastically affected by the sublethal insecticide exposure, which impaired its population growth and thus favored its heterospecific competitor *R. dominica*.

**Figure 4:**
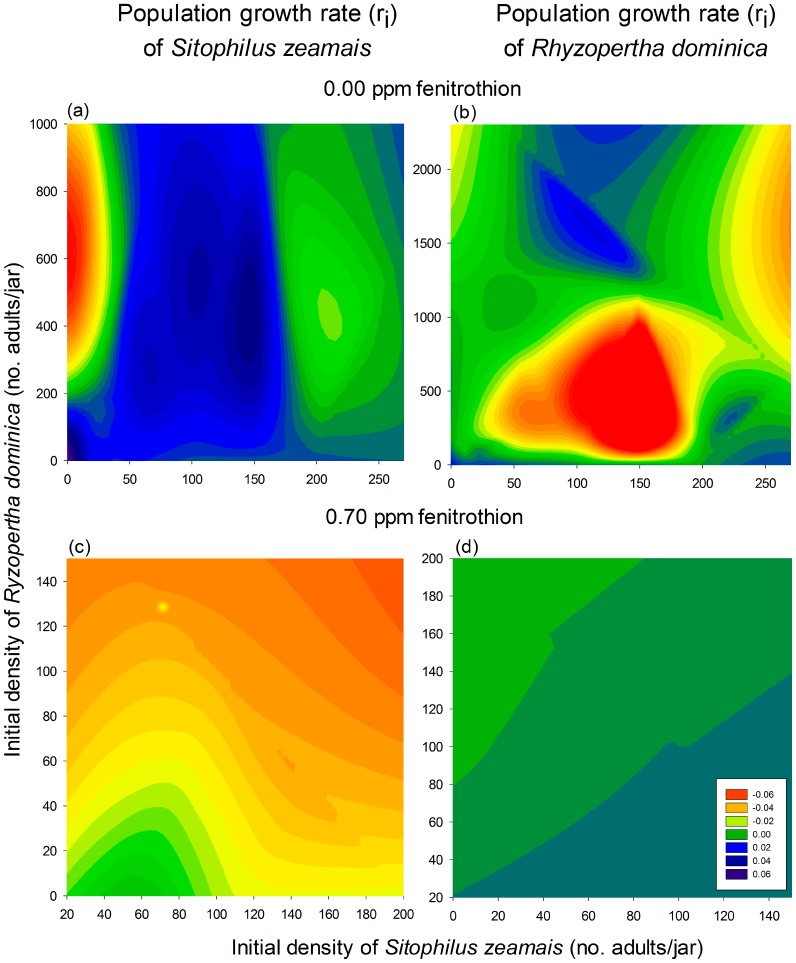
Filled contour plots exhibiting the effect of conspecific and heterospecific densities on the population growth of two species of stored grain beetles (*Sitophilus zeamais* and *Rhyzopertha dominica*) reared on maize grains that were untreated or treated with 0.7 ppm of the organophosphate insecticide fenitrothion.

The even and uneven density combinations of *S. zeamais* and *R. dominica* following the additive series indicated the prevalence of the former species under interspecific competition in uncontaminated maize grains (Fig. 4). *R. dominica* is more severely affected by both conspecific and heterospecific competitors than *S. zeamais*, which prevailed as the dominant species in maize grains free of insecticide residues. However, the pattern of species prevalence shifted when the insecticide fenitrothion was used to treat the grains. Under fenitrothion exposure, *S. zeamais* was more strongly affected by not only conspecific competition but also heterospecific competition. As a result, *R. dominica* became dominant.

## Discussion

Competitive interactions negatively affect all of the competitors. The frequent perception of competition as an intermittent phenomenon may compromise the recognition of its potential importance [3], [5]–[7]. However, competition is common and is highly important among grain beetles that are internal feeders and share the same resource throughout their development [28]–[32], [39]. Environmental disturbances, such as insecticide application and the resulting seed contamination, potentially interfere with the availability and suitability of the shared resources, affecting competitive interactions and their expected outcome. Curiously, insecticide-mediated competitive interactions have seldom been investigated, and the few previous investigations have focused primarily on intraspecific competition [16], [40]–[42]. In this study, we tested the effect of insecticide disturbance as a mediator of intra- and interspecific competition in two cereal grain beetle (pest) species characterized by internal feeding. These species do not leave the individual grain until they emerge as adults.

Toxic compounds, particularly insecticides, are not static mediators of ecological interactions. They change with time due to their environmental degradation, they potentially exhibit direct and indirect effects on the exposed organisms, their effects on these organisms may be lethal and/or sublethal, and they have differential effects on exposed individuals of a given species or even populations within a species [18]–[21], [43]. The expected result of our competition studies with the cereal grain beetles *S. zeamais* and *R. dominica* was an alleviation of intra- and interspecific competition that would allow better co-existence of the two pest species. This expectation is justified because the insecticide used, the organophosphate fenitrothion, is recommended for maize grain protection under conditions in which both species co-occur and is, therefore, effective in rapidly reducing the populations of these insects in treated maize grains [33], [35]. However, the insecticide fenitrothion enhanced the crowding effect, intensifying intraspecific competition, especially in *S. zeamais*. The insecticide also produced a shift in competitive dominance, which resulted in a shift in prevalence between the co-occurring cereal beetle species.

Although an insecticide kills most of the individuals of the target species, it will most likely enhance the probability of survival of the remaining individuals by increasing the relative amount of resources available for their development, thus producing more rapid population growth. This phenomenon is termed “competition release” and has been proposed and successfully tested in amphibians and mosquitoes subjected to intraspecific competition [41], [42]. Competition release is a potential cause of pest resurgence (i.e., an increase in the abundance of the targeted arthropod pest species to a level exceeding that of uncontrolled populations following insecticide (or acaricide) application), but never tested [44], [45]. Such insecticide-mediated release from competitive stress was not observed in our study of intraspecific competition in grain beetles.

The insecticide enhanced the effect of insect density, particularly for the maize weevil *S. zeamais*, further impairing population growth at high densities increasing the efficacy of this toxic compound. Therefore, insecticide susceptibility increased with intraspecific competition, as also reported for a microcrustacean, *Daphnia* spp. [46]. As potential selection under crowded conditions may interfere with the response to intraspecific competition in mosquitoes [47], low density (or crowded conditions) may be favored by cereal beetles, either by substrate selection (for egg-laying and/or feeding, depending on the species) or by direct larval interference within the grain [28], [29], [32]. Evidence for this effect exists, at least in *S. zeamais*, with direct and potentially indirect effects favoring larval competition and survival at low densities [29], [32]. Most likely, these effects produce fitter individuals that can tolerate a higher insecticide exposure. In addition, high densities (and intraspecific competition) can delay the recovery of the population structure under insecticide exposure due to high adult mortality and the potential impairment of juvenile development, as observed in *Daphnia magna* Straus [48]. However, the insecticide used in our study primarily affects mainly adults. Therefore, the high adult mortality would be the most likely reason that the insecticide enhances the negative impact of intense competition.

The effect of insecticide exposure on competitive interaction extends beyond intraspecific competition, affecting interspecific competition as well. Competing species may coexist, or one may exclude the other, depending on their intrinsic life history traits and initial densities [49], [50]. These characteristics can be affected by insecticide activity. For instance, asymmetry may exist between competing species, with a dominant (prevailing) competitor displacing or at least containing the other (at smaller densities), but the introduction of an agent of stress in the system may alter this condition. The competing cereal beetles *S. zeamais* and *R. dominica* investigated in our study exhibit an asymmetry in their interaction, with the former species prevailing over the latter in maize grains and excluding it at high densities. However, insecticide disturbance can shift the outcome of competition, allowing *R. dominica* to prevail over *S. zeamais* under intermediate insecticide exposure, causing the eventual exclusion of the weevil. Evidence for such effects has been reported in frog tadpoles exposed to the carbamate insecticide carbaryl [51], in *Daphnia* and *Culex* larvae exposed to the pyrethroid insecticide fenvalerate [52], and in *Aedes* mosquitoes exposed to the organophosphate malathion [27]. In fact, habitat contamination by insecticide residues can alter species colonization patterns [53].

The competitive outcomes observed in our study with cereal grain beetles appear to give credence to the intermediate disturbance hypothesis, which predicts that intermediate levels of disturbance will maximize species diversity while reducing the proportional abundance of competitively dominant species [10], [11], [54]. This hypothesis has been the subject of recent controversy aimed at its broad disturbance-diversity predictions [55], [56], but its predictions have seldom been explored in the context of competitive interactions among animals. The reason for this lack of attention is, most likely, that the intermediate disturbance hypothesis was first conceived and generally explored in studies of plant communities (particularly tropical forests) and coral reefs [54], [56].

Insecticide disturbance appears well suited for consideration in terms of the intermediate disturbance hypothesis. This hypothesis will assist us to understand competitive interactions in cereal grain beetles. High insecticide doses and rates of application are likely to suppress one, if not both, competing beetle species. This principle is illustrated in our experiments by the suppression of *S. zeamais* in maize grains treated with 5.0 ppm fenitrothion, a dose that is nearly 2 times lower than the range recommended for maize protection [35]. Such outcome may result in outbreaks of the insecticide-favored pest species in contaminated environments, a phenomenon frequently reported but whose causes are poorly known and usually not tested [44], [45], [57]. Excessively low insecticide doses will not interfere significantly with the competitive interaction, and the dominant species should prevail, as suggested by the results obtained with 0.2 ppm fenitrothion in our study. However, intermediate doses of fenitrothion (ranging from 0.5–0.7 ppm) significantly compromised the population growth of *S. zeamais* and competitively favored *R. dominica*, which is inherently more tolerant to this insecticide. This shift in the competition outcome may also result in the outbreak of the insecticide-favored pest species – *R. dominica* in the case of the present case.

This higher tolerance of *R. dominica* to fenitrothion may be due to its physiology and/or behavior (e.g., adults of this species are less active than *S. zeamais*, remaining longer within the grain and potentially minimizing insecticide exposure). Regardless of the tolerance mechanism, intermediate doses of fenitrothion shifted the species dominance, minimizing the likelihood of competitive exclusion of *R. dominica* by *S. zeamais*. Therefore, such an increase in diversity with intermediate disturbance provides support for the intermediate disturbance hypothesis as a potential explanation for the observed outcome. This hypothesis will, most likely, prove useful in studies of competitive interactions under toxic stress agents. As relatively low insecticide residues persist in the environment for longer periods due to their natural rate of degradation, they may be important because they play unrecognized roles in favoring the co-occurrence of multiple pest species. This phenomenon, with potential management consequences as a potential cause of pest outbreaks, has been largely neglected to date and deserves careful attention.

## Supporting Information

Material S1 **Raw data of** **Fig. 4** **.**
(PDF)

## References

[1] Gause GF, Witt AA (1935) Behavior of mixed populations and the problem of natural selection. Amer Nat 69: 596–60.

[2] Crombie AC (1947) Interspecific competition. J Anim Ecol 16: 44–73.

[3] Park T (1948) Interspecies competition in population of *Tribolium confusum* Duval and *Tribolium castaneum* Herbst. Ecol Monogr 18: 265–307.

[4] Schoener TW (1982) The controversy over interspecific competition. Am Sci 70: 586–595.

[5] Ayala FJ (1969) Experimental invalidation of the principle of competitive exclusion,. Nature 224: 1076–1079. 5353714 10.1038/2241076a0

[6] Wiens JA (1977) On competition and variable environments. Am Sci 65: 590–597.

[7] Wiens JA (1986) Spatial scale and temporal variation in studies of scrubsteppe birds. In: Diamond JM, Case TJ, editors. Community ecology. New York: Harper & Row. pp.154–172.

[8] Birch LC (1953) Experimental background to the study of the distribution and abundance of insects. III. The relation between innate capacity for increase and survival of different species of beetles living together on the same food. Evolution 7: 136–44.

[9] Birch LC (1957) The meanings of competition. Am Nat 91: 5–18.

[10] Connell JH (1978) Diversity in tropical rain forests and coral reefs – high diversity of trees and corals is maintained only in a non-equilibrium state. Science 199: 1302–1310. 17840770 10.1126/science.199.4335.1302

[11] Shea K, Roxburgh SH, Rauschert ES (2004) Moving from pattern to process: Coexistence mechanisms under intermediate disturbance regimes. Ecol Lett 7: 491–508.

[12] Pickett STA, White PS (1985) Natural disturbance and patch dynamics: an introduction. In: Pickett STA, White PS, editors. The Ecology of Natural Disturbance and Patch Dynamics. Orlando: Academic Press. pp 3–13.

[13] Reitz SR, Trumble JT (2002) Competitive displacement among insects and arachnids. Annu Rev Entomol 47: 435–465. 11729081 10.1146/annurev.ento.47.091201.145227

[14] Witman JD (1987) Subtidal coexistence: storms, grazing, mutualism, and the zonation of kelps and mussels. Ecol Monogr 57: 167–187.

[15] Liess M, Von Der Ohe PC (2005) Analyzing effects of pesticides on invertebrate communities in streams. Environ Toxicol Chem 24: 954–965. 15839571 10.1897/03-652.1

[16] Beketov MA, Liess M (2006) The influence of predation on the chronic response of *Artemia* sp. populations to a toxicant. J Appl Ecol 43: 1069–1074. 18784796 10.1111/j.1365-2664.2006.01226.x PMC2368765

[17] Pestana JL, Loureiro S, Baird DJ, Soares AM (2009) Fear and loathing in the benthos: Responses of aquatic insect larvae to the pesticide imidacloprid in the presence of chemical signals of predation risk. Aquat Toxicol 93: 138–149. 19477535 10.1016/j.aquatox.2009.04.008

[18] Haynes KF (1988) Sublethal effects of neurotoxic insecticides on insect behavior. Annu Rev Entomol 33: 149–68. 3277528 10.1146/annurev.en.33.010188.001053

[19] Stark JD, Banks JE (2003) Population-level effects of pesticides and other toxicants on arthropods. Annu Rev Entomol 48: 505–519. 12221038 10.1146/annurev.ento.48.091801.112621

[20] Desneux N, Decourtye A, Delpuech JM (2007) The sublethal effects of pesticides on beneficial arthropods. Annu Rev Entomol 52: 81–106. 16842032 10.1146/annurev.ento.52.110405.091440

[21] Guedes RNC, Magalhães LC, Cosme LV (2009) Stimulatory sublethal response of a generalista predator to permethrin: hormesis, hormoligosis, or homeostatic regulation? J Econ Entomol 102: 170–176. 19253633 10.1603/029.102.0124

[22] Wissinger S (1989) Seasonal variation in the intensity of competition and predation among dragonfly larvae. Ecology 70: 1017–1027.

[23] Stark JD, Tanigoshi L, Bounfour M, Antonelli A (1997) Reproductive potential: its influence on the susceptibility of a species to pesticides. Ecotoxicol Environ Saf 37: 273–279. 9378095 10.1006/eesa.1997.1552

[24] Oliveira EE, Guedes RNC, Totola MR, De Marco P Jr (2007) Competition between insecticide-susceptible and -resistant populations of the maize weevil, *Sitophilus zeamais* . Chemosphere 69: 17–24. 17570459 10.1016/j.chemosphere.2007.04.077

[25] Sun D-B, Liu Y-Q, Qin L, Xu J, Li F-F, et al (2013) Competitive displacement between two invasive whiteflies: insecticide application and host plant effects. Bull Entomol Res 103: 344–353. 23458717 10.1017/S0007485312000788

[26] Gao Y, Reitz SR, Wei Q, Yu W, Zhang Z, et al (2014) Local crop planting systems enhance insecticide-mediated displacement of two invasive leafminer fly. PLoS ONE 9 (3) e92625. 24651465 10.1371/journal.pone.0092625 PMC3961394

[27] Kesavaraju B, Afify A, Gaugler R (2013) Strain specific differences in intraspecific competition in *Aedes albopictus* (Diptera: Culicidae). J Med Entomol 49: 988–992. 10.1603/me11245 23025178

[28] Smith RH, Lessells CM (1985) Ovipositiion, ovicide and larval competition in granivorous insects. In: Sibly RM, Smith RH, editors. Behavioural Ecology: Ecological Consequences of Adaptive Behaviour. London: Blackwell. pp. 423–448.

[29] Smith RH (1991) Genetic and phenotypic aspects of life-history evolution in animals. Adv Ecol Res 21: 63–120.

[30] Colegrave N (1994) Game theory models of competition in closed systems: asymmetries in fighting and competitive ability. Oikos 71: 499–505.

[31] Guedes RNC, Guedes NMP, Smith RH (2007) Larval competition within seeds: From the behaviour process to the ecological outcome in the seed beetle *Callosobruchus maculatus* . Austral Ecol 32: 697–707.

[32] Guedes NMP, Guedes RNC, Campbell JF, Throne JE (2010) Contest behavior of maize weevil larvae when competing within seeds. Anim Behav 79: 281–289.

[33] Rees DJ (1996) Coleoptera. In: Subramanyam Bh, Hagstrum DW, editors. Integrated Management of Insects in Stored Products. New York: Marcel Dekker. pp. 1–39.

[34] Guedes RNC, Guedes NMP, Rosi-Denadai CA (2011) Sub-lethal effects of insecticides on stored-product insects: current knowledge and future needs. Stewart Postharv Rev 7: 1–5.

[35] MAPA [Ministério da Agricultura, Agropecuária e Abastecimento] (2014) AGROFIT: Sistema de Agrotóxicos Fitossanitários. Available: http://extranet.agricultura.gov.br/agrofit_cons/principal_agrofit_cons. Accessed 3 April 2014.

[36] Walthall WK, Stark JD (1997) Comparison of two population-level ecotoxicological endpoints: the intrinsic (r_m_) and instantaneous (r_i_) rates of increase. Environ Toxicol Chem 16: 1068–1073.

[37] SAS Institute (2008) SAS/STAT User's Guide. Cary, NC, USA: SAS Institute.

[38] Forbes VE, Calow P (1999) Is the per capita rate of increase a good measure of population-level effects in ecotoxicology? Environ Toxicol Chem 18: 1544–1556.

[39] Vilca Mallqui KS, Oliveira EE, Guedes RNC (2013) Competition between the bean weevils *Acanthoscelides obtectus* and *Zabrostes subfasciatus* in common beans. J Stored Prod Res 55: 32–35.

[40] Linke-Gamenick I, Forbes VE, Sibly RM (1999) Density-dependent effects of a toxicant on life-history traits and population dynamics of a *Capitellid polychaete* . Mar Ecol - Prog Ser 184: 139–148.

[41] Boone MD, Semlitsch RD (2002) Interactions of an insecticide with competition and pond drying in amphibian communities. Ecol Appl 12: 307–316.

[42] Muturi EJ, Costanzo K, Kesavaraju B, Alto BW (2011) Can pesticides and larval competition alter susceptibility of *Aedes* mosquitoes (Diptera: Culicidae) to arbovirus infection? J Med Entomol 48: 429–436. 21485385 10.1603/me10213

[43] Guedes RNC, Cutler GC (2014) Insecticide-induced hormesis and arthropod pest management. Pest Manag Sci 70: 690–697. 24155227 10.1002/ps.3669

[44] Ripper WE (1956) Effect of pesticides on balance of arthropod populations. Annu Rev Entomol 1: 403–438.

[45] Hardin MR, Benrey B, Colt M, Lamp W, Roderick GK, et al (1995) Arthropod pest resurgence: An overview of potential mechanisms. Crop Prot 14: 3–18.

[46] Knillmann S, Stampfli NC, Beketov MA, Liess M (2012) Intraspecific competition increases toxicant effects in outdoor pond microcosms. Ecotoxicology 21: 1857–1866. 22572781 10.1007/s10646-012-0919-y

[47] Kesavaraju B, Afify A, Gaugler R (2013) Strain specific differences in intraspecific competition in *Aedes albopictus* (Diptera: Culicidae). J Med Entomol 49: 988–992. 10.1603/me11245 23025178

[48] Liess M, Foit K (2010) Intraspecific competition delays recovery of population structure. Aquat Toxicol 97: 15–22. 20053462 10.1016/j.aquatox.2009.11.018

[49] Lotka AJ (1925) Elements of Physical Biology. Baltimore: Williams & Wilkins.

[50] Lotka AJ (1932) The growth of mixed populations: Two species competing for a common food supply. J Wash Acad Sci 22: 461–69.

[51] Mills NE, Semlitsch RD (2004) Competition and predation mediate the indirect effects of an insecticide on southern leopard frogs. Ecol Appl 14: 1041–1054.

[52] Foit K, Kaske O, Liess M (2012) Competition increases toxicant sensitivity and delays the recovery of two interacting populations. Aquat Toxicol 106–107: 25–31. 10.1016/j.aquatox.2011.09.012 22057252

[53] Vonesh JR, Kraus JM (2009) Pesticide alters habitat selection and aquatic community composition. Oecologia 160: 379–385. 19252931 10.1007/s00442-009-1301-5

[54] Wilkinson DM (1999) The disturbing history of intermediate disturbance. Oikos 84: 145–147.

[55] Fox JW (2013) The intermediate disturbance hypothesis should be abandoned. Trends Ecol Evol 28: 86–92. 22981468 10.1016/j.tree.2012.08.014

[56] Sheil D, Burslem DFRP (2013) Defining and defending Connell's intermediate disturbance hypothesis: A response to Fox. Trends Ecol Evol 28: 571–572. 23953996 10.1016/j.tree.2013.07.006

[57] Cordeiro EMG, Moura ILT de, Fadini MAM, Guedes RNC (2013) Beyond selectivity: Are behavioral avoidance and hormesis likely causes of pyrethroid-induced outbreaks of the southern red mite *Olygonychus ilicis*? Chemosphere 93: 1111–1116. 23830118 10.1016/j.chemosphere.2013.06.030

