# Supplementary material for: Insecticide-Mediated Shift in Ecological Dominance between Two Competing Species of Grain Beetles
Source: PLoS One. 2014 Jun 24;9(6):e100990. doi: 10.1371/journal.pone.0100990 (PMC4069159; doi:10.1371/journal.pone.0100990)
Supplement: Material S1 — Raw data of Fig. 4 . (PDF) [file pone.0100990.s001.pdf]

Fig. 4a

S. zeamais	R. dominica	S. zeamais ri
20	0	0.0224
20	0	0.0319
20	0	0.0273
20	0	0.0265
50	0	0.0224
50	0	0.0225
50	0	0.0225
50	0	0.0141
100	0	0.017
100	0	0.0147
100	0	0.0142
100	0	0.0133
150	0	0.0114
150	0	9.88E-03
150	0	0.0189
150	0	0.0159
200	0	0.0144
200	0	0.0111
200	0	0.0106
200	0	9.84E-03
5	100	0.0374
10	100	0.0319
15	100	0.0239
20	1400	0.025
40	2300	0.0212
60	800	0.0214
150	1200	9.03E-03
200	200	-2.34E-03
270	600	7.26E-03
200	0	0.0178
200	0	0.0163
200	0	0.0169
150	50	0.0221
150	50	0.0208
150	50	0.0217
100	100	0.0229
100	100	0.026
100	100	0.0231
50	150	0.0271
50	150	0.0311
50	150	0.0286
0	200	0
0	200	0
0	200	0

Fig. 4b

S. zeamais	R. dominica	R. dominica ri
20	0	0.028
20	0	7.70E-03
20	0	0.0138
20	0	0.0136
50	0	0.0185
50	0	0.0107
50	0	0.0128
50	0	0.0109
100	0	4.80E-03
100	0	0.0108
100	0	7.81E-03
100	0	6.28E-03
150	0	6.73E-03
150	0	5.94E-03
150	0	8.24E-03
150	0	7.13E-03
200	0	0.0128
200	0	4.65E-03
200	0	8.56E-03
200	0	5.36E-03
5	100	2.57E-03
10	100	-3.05E-03
15	100	1.93E-03
20	1400	-5.22E-03
40	2300	-4.24E-03
60	800	-0.0115
150	1200	-2.56E-03
200	200	-3.50E-03
270	600	-0.0152
200	0	0
200	0	0
200	0	0
150	50	-0.0358
150	50	-0.0218
150	50	-0.0522
100	100	-0.0204
100	100	-0.0241
100	100	-0.024
50	150	-4.77E-03
50	150	-0.0271
50	150	-0.0391
0	200	2.67E-03
0	200	5.19E-03
0	200	3.26E-03

Fig. 4c

S. zeamais	R. dominica	S. zeamais ri
200	0	-0.0232
200	0	-0.0117
150	50	-0.0409
150	50	-0.0483
150	50	-0.0326
100	100	-0.0429
100	100	-0.0337
100	100	-0.0375
50	150	-0.0522
50	150	-0.0429
50	150	-0.0429
20	0	-0.0256
20	0	-6.64E-03
20	0	0.011
20	0	-0.0256
50	0	-2.48E-03
50	0	2.57E-03
50	0	-0.0204
50	0	0.0177
100	0	-0.0281
100	0	3.17E-03
100	0	-7.70E-03
100	0	-0.0358
150	0	-0.0168
150	0	-0.0264
150	0	-0.0264
150	0	
200	0	-0.0123
200	0	-0.0589
200	0	-0.0152
200	0	-0.0128

Fig 4d

S. zeamais	R. dominica	R. dominica ri
150	50	0.0108
150	50	5.93E-03
150	50	0.0153
100	100	9.83E-03
100	100	3.70E-03
100	100	0.0113
50	150	6.70E-03
50	150	4.29E-03
50	150	1.75E-03
0	200	5.80E-03
0	200	1.33E-03
0	20	-1.17E-03
0	20	0.0142
0	20	0.011
0	20	8.24E-03
0	50	3.25E-03
0	50	8.56E-03
0	50	6.03E-03
0	50	6.28E-03
0	100	1.93E-03
0	100	3.08E-03
0	100	1.55E-03
0	100	5.29E-03
0	150	3.52E-03
0	150	4.05E-03
0	150	1.96E-03
0	150	7.86E-04
0	200	7.00E-04
0	200	1.55E-03
0	200	9.06E-04
0	200	-1.68E-04
